# Downregulated GTCPH I/BH4 Pathway and Decreased Function of Circulating Endothelial Progenitor Cells and Their Relationship with Endothelial Dysfunction in Overweight Postmenopausal Women

**DOI:** 10.1155/2018/4756263

**Published:** 2018-07-01

**Authors:** Ying Luo, Zhenhua Huang, Jinli Liao, Zhihao Liu, Xiaopeng Li, Shun Yao, Hao He, Dajun Hu, Zi Ren, Haitao Zeng, Quanneng Yan, Hong Zhan

**Affiliations:** ^1^Department of Geriatric Medicine, Xiangya Hospital, Central South University, Changsha 410008, China; ^2^Department of Emergency Medicine, The First Affiliated Hospital, Sun Yat-sen University, Guangzhou 510080, China; ^3^Guangzhou Beijing Community Health Service Center, Guangzhou 510080, China; ^4^Department of Cardiology, The First Affiliated Hospital, Sun Yat-sen University, Guangzhou 510080, China; ^5^Department of Cardiology, Nanhai Hospital, Southern Medical University, Foshan 528200, China; ^6^Center for Reproductive Medicine, The Sixth Affiliated Hospital, Sun Yat-sen University, Guangzhou 510080, China; ^7^Department of Cardiology, Heart Center, Zhujiang Hospital, Southern Medical University, Guangzhou 510280, China

## Abstract

Endothelial progenitor cells (EPCs) have endogenous endothelium-reparative potential, but obesity impairs EPCs. Overweight premenopausal women have a normal number of circulating EPCs with functional activity, but whether EPCs in overweight postmenopausal women can repair obesity-related endothelial damage requires further investigation. For this purpose, we examined the function and number of circulating EPCs, evaluated vascular endothelial function, and explored the underlying mechanism. Compared with normal weight or overweight age-matched men, postmenopausal women (overweight or normal weight) had a diminished number of circulating EPCs and impaired vascular endothelial function, as detected by flow-mediated dilatation. Moreover, GTCPH I expression and the nitric oxide level in overweight postmenopausal women and men were significantly decreased. Together, our findings demonstrate that the number or function of circulating EPCs and endothelial function, which is partially regulated by the GTCPH I/BH4 signaling pathway, is not preserved in overweight postmenopausal women. The GTCPH I/BH4 pathway in circulating EPCs may be a potential therapeutic target for endothelial injury in overweight postmenopausal women.

## 1. Introduction

Overweight and obesity are associated with an increased risk of cardiovascular diseases, such as stroke, coronary atherosclerosis, congestive heart failure, and arrhythmias [1–7]. Abundant data have demonstrated that overweight or obesity may contribute to endothelial damage and dysfunction [1, 2, 6–9], which are believed to be involved in the pathogenesis and progression of cardiovascular diseases [10]. Therefore, it is urgent to identify a method to restore endothelial dysfunction in overweight or obese individuals.

Circulating endothelial progenitor cells (EPCs), which are derived from the bone marrow, maintain endothelial integrity and improve vascular endothelial function [10, 11]. It may be a surrogate biologic marker for cumulative cardiovascular risk and affect the progression of cardiovascular disease [12], and it predicts the occurrence of cardiovascular events and death from cardiovascular causes [13]. Previous studies have suggested that EPCs are closely associated with flow-mediated dilatation (FMD), which noninvasively assesses endothelium-dependent function by the brachial artery using high-resolution ultrasound [14]. Recent studies have shown that EPCs with a decreased number and dysfunction were observed in patients with cardiovascular risk factors, such as overweight or obesity, those with hypertension, and those who smoke and/or drink [15–17]. Estrogen plays a crucial role in the prevention of vascular endothelial injury and dysfunction. It is consistent with the view that postmenopausal women have an increased prevalence of cardiovascular disease and obesity, and it may be related to that the benefit from estrogen on endothelial repair will gradually weaken in the postmenopausal women [11]. Some researchers believe that estrogen induces EPC mobilization from the bone marrow to promote vessel growth and enhance the repair of endothelial injury [18]. Previous studies revealed that prehypertensive premenopausal women had a normal number, and functional activity, of EPCs [10, 15, 19, 20]. The incidence of cardiovascular diseases in premenopausal women is lower than that in age-matched men; however, postmenopausal women are at an increasingly higher risk than premenopausal women [21]. These results indicate that estrogen may prevent cardiovascular disease. Researchers have also found that estrogen deficiency decreases the capacity of EPCs in endothelial repair and contributes to age-matched vascular injury in women [19, 20]. Therefore, endothelial injury could increase the risk of cardiovascular diseases in postmenopausal women [11, 22].

Our previous study showed that the functional activity of circulating EPCs was diminished in overweight or obese men but not in overweight or obese premenopausal women. However, whether this favorable effect exists in overweight postmenopausal women is not clear. We hypothesized that the number and functional activity (migration and proliferation) of circulating EPCs in overweight premenopausal women or men may differ from that in overweight postmenopausal women. In addition, nitric oxide (NO), vascular endothelial growth factor (VEGF), granulocyte macrophage colony-stimulating factor (GM-CSF), tumor necrosis factor-alpha (TNF-*α*), and interleukin-6 (IL-6) play important roles in regulating circulating EPCs [23–28]. Tetrahydrobiopterin (BH4) is a required cofactor for the synthesis of NO by eNOS, and its level is determined by the activity guanosine triphosphate cyclohydrolase I (GTPCH I) [29]. It was reported that GTPCH I may enhance endothelial NO synthase coupling and increase the number and function of circulating EPCs after vascular injury [30]. To test our hypothesis, we measured the number and functional activity of circulating EPCs in overweight postmenopausal women and men, evaluated the level of NO, VEGF, GM-CSF, TNF-*α*, and IL-6 in plasma or EPC culture medium, investigated the expression of the GTCPH I/BH4 signaling pathway in circulating EPCs, and elucidated the mechanism underlying the altered endothelial repair capacity in overweight postmenopausal women.

## 2. Patients and Methods

### 2.1. Patients and Subjects

Twenty normal weight postmenopausal women, 20 overweight postmenopausal women, 20 normal weight men, and 20 overweight men were recruited. Postmenopausal women, in brief, were after 42–58 years of age and at least 6 months and no more than 36 months from last menstrual period [31]. All subjects with a smoking habit, cardiovascular, hypertension, or metabolic disease, inflammatory disorders, and diabetes were excluded because these factors may influence the function and number of EPCs. The experimental protocol was approved by the Ethical Committees of our hospitals.  Table 1 shows the baseline characteristics of four groups of subjects. Peripheral blood samples were used to determine the number of EPCs, fasting plasma glucose (FPG), aspartate amino transferase (AST), alanine transaminase (ALT), blood urea nitrogen (BUN), high-density lipoprotein (HDL) cholesterol, triglyceride (TG), low-density lipoprotein (LDL) cholesterol, serum creatinine (Cr), and total cholesterol.

### 2.2. EPC Culture and Detecting the Number of Circulating EPCs by Cell Culture Assays and Flow Cytometry

EPC culture and the number of EPCs were evaluated as described in the previous studies [14, 23, 32]. In brief, peripheral blood mononuclear cells were isolated by Ficoll density gradient centrifugation, and it was cultured in endothelial cell basal medium-2 (EBM-2) (Clonetics, San Diego, CA, USA).

The number of circulating EPCs was detected by CD34^+^/KDR^+^ cells per 100 peripheral blood mononuclear cells. The circulating EPCs were isolated and cultured. After one week, the number of cultured EPCs was detected by DiI-acLDL/lectin double-positive cells as previously reported and counted manually by two independent observers blinded to the study.

### 2.3. EPC Migration and Proliferation Assays

EPC migration and proliferation assays were performed as previously described [23, 32]. In brief, EPCs were cultured for one week and were counted. Then, 2 × 10^4^ EPCs were placed in the upper chamber of a modified Boyden chamber. After 24 h, cell nuclei were stained with DAPI. Cells migrating into the lower chamber were counted by microscope. EPC proliferation was determined by 3-(4,5-dimethylthiazol-2-yl)-2,5-diphenyltetrazolium bromide (MTT) (5 g/L; Fluka Co. Product) assay. The OD value was measured at 490 nm [15].

### 2.4. Measurement of NO, GM-CSF, VEGF, IL-6, and TNF-*α* Levels in Plasma and Secretion by EPCs

NO, VEGF, and GM-CSF levels in plasma and secretion by EPCs were evaluated as previously described [23]. We used ELISA kits to determine plasma concentrations of interleukin-6 (IL-6; R&D Systems, Minneapolis, MN, USA) and tumor necrosis factor-*α* (TNF-*α*; R&D Systems, Minneapolis, MN, USA) [33].

### 2.5. Flow-Mediated Dilatation

FMD was performed according to the previous report [14, 15]. In brief, brachial artery FMD was measured by high-resolution ultrasonography using a 5–12 MHz linear transducer on an HDI 5000 system (Washington, USA). The brachial artery was studied 20 to 100 mm proximal to the antecubital fossa in supine participants after 15 min rest. Pressure in an upper-forearm sphygmomanometer cuff was raised to 250 mmHg for 5 min and FMD calculated as the percentage increase in mean diastolic diameter after reactive hyperaemia 55 to 65 s after deflation to baseline.

### 2.6. Western Blot Analysis and Intracellular Tetrahydrobiopterin Measurement

EPC proteins were harvested by cell lysis buffer (Cell Signaling Technology Inc., Danvers, Massachusetts, USA). The following antibodies were used: rabbit anti-GTPCH I and anti-*β*-actin (1 : 1000; Santa Cruz Technology Inc.). The intensity of immunoreactive bands was analyzed, and results were expressed as the ratio of proteins of GTPCH I to proteins of *β*-actin in human EPCs.

Intracellular BH4 level was measured by high-performance liquid chromatography with florescence detection after iodine oxidation in acidic or alkaline conditions. BH4 concentrations, expressed as pmol/mg protein, were calculated by subtracting dihydrobiopterin (BH2) and oxidized biopterin from the total biopterins [30].

### 2.7. Statistical Analysis

The statistic software used was SPSS v11.0 (SPSS Inc., Chicago, IL, USA). All data are presented as the mean ± standard deviation (SD). Comparisons between the four groups were analyzed by two-factor analysis of variance (sex and status of normal weight or overweight). When indicated by a significant *F* value, a post hoc test using the Newman-Keuls method identified significant differences among mean values. Univariate correlations were calculated using Pearson's coefficient (*r*). Statistical significance was assumed if a null hypothesis could be rejected at *p* < 0.05.

## 3. Results

### 3.1. Baseline Characteristics

As shown in Table 1, the four groups were similar in terms of age. Height and weight were significantly higher in age-matched men compared with postmenopausal women (*p* < 0.05). Weight and body mass index were higher in overweight postmenopausal women and overweight men than in normal weight postmenopausal women and men (*p* < 0.05).

FMD was lower in overweight postmenopausal women and men than in normal weight men and postmenopausal women (*p* < 0.05). However, there was no difference in FMD between postmenopausal women and age-matched men (*p* > 0.05). There were no differences in the heart rate, diastolic blood pressure, systolic blood pressure, or levels of AST, ALT, BUN, Cr, LDL, TC, HDL, TG, FPG, or estradiol among the four groups (*p* > 0.05).

### 3.2. Number and Function of Circulating EPCs

**Figure 1:**
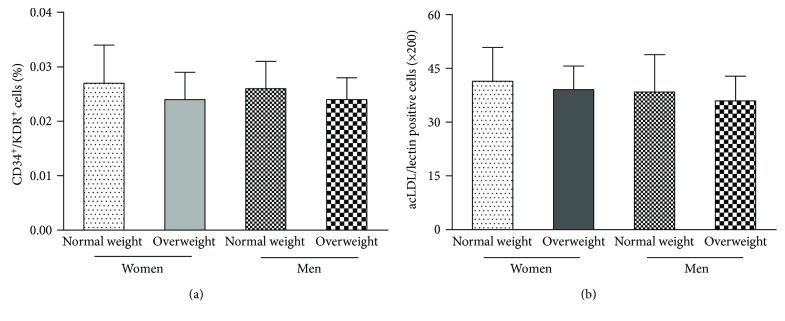
The number of circulating endothelial progenitor cells (EPCs) was evaluated as follows: by (a) fluorescence-activated cell sorting analysis and (b) phase-contrast fluorescent microscopy. The number of circulating EPCs in normal weight and overweight men was similar to that in normal weight and overweight postmenopausal women. The EPC number in overweight men and postmenopausal women was also similar to that in normal weight men and postmenopausal women. Data are given as the mean ± standard deviation (SD). ^∗^*p* < 0.05 versus normal weight.

Figure 1 shows the number of circulating EPCs in the four groups. The number of circulating EPCs in normal weight and overweight men was similar to that in normal weight and overweight postmenopausal women (*p* > 0.05).

**Figure 2:**
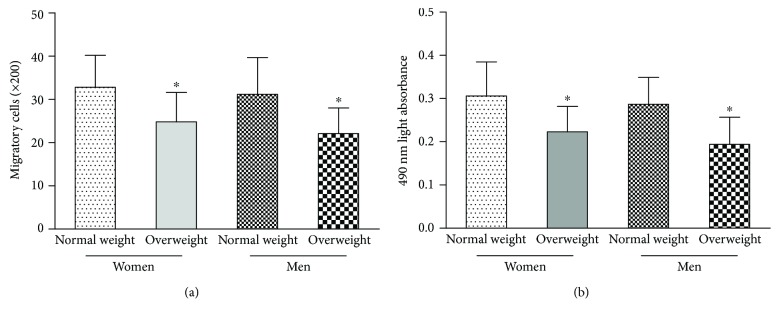
The activity of circulating EPCs was evaluated as follows. The migratory (a) and proliferative (b) activities of circulating EPCs in normal weight and overweight men were similar to those in normal weight and overweight postmenopausal women. EPC function was lower in overweight men and postmenopausal women than in normal weight men and postmenopausal women. Data are given as the mean ± SD. ^∗^*p* < 0.05 versus normal weight.

As shown in Figure 2, the migration (a) and proliferation (b) of circulating EPCs in normal weight men were similar to those in normal weight postmenopausal women (*p* > 0.05). In addition, the migration (a) and proliferation (b) of circulating EPCs in overweight men were similar to those in overweight postmenopausal women (*p* > 0.05). EPC function (migration and proliferation) was lower in overweight men than in normal weight men and postmenopausal women (*p* < 0.05).

### 3.3. Plasma NO, GM-CSF, VEGF, IL-6, and TNF-*α* Levels

**Figure 3:**
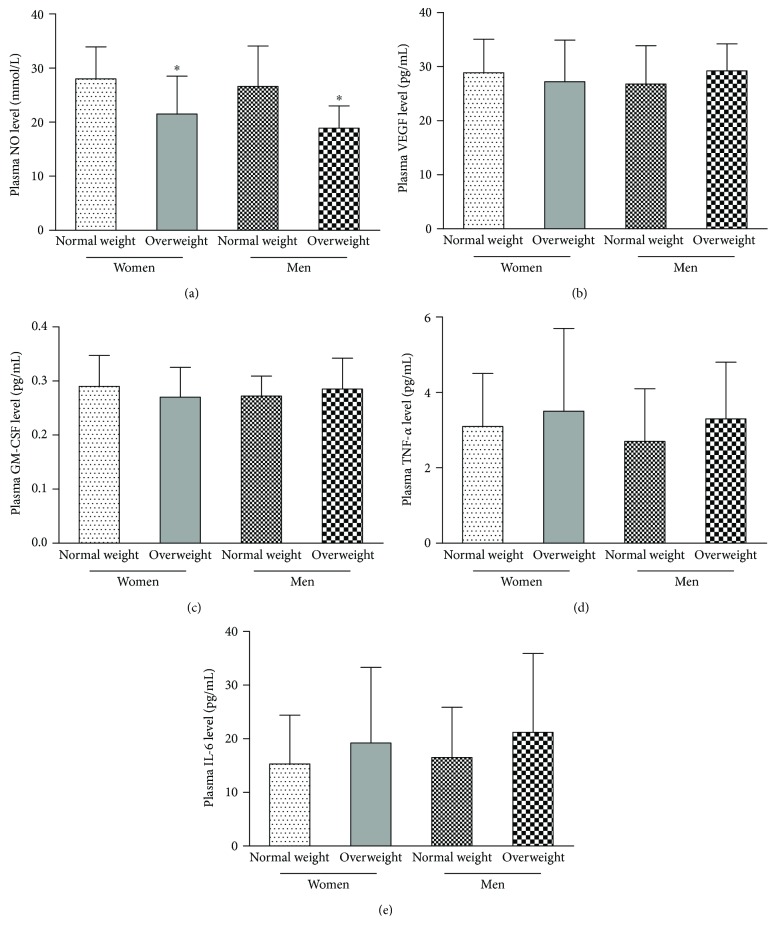
Plasma NO, VEGF, GM-CSF, TNF-*α*, and IL-6 levels were evaluated as follows. (a) The plasma NO level in normal weight and overweight men was similar to that in normal weight and overweight postmenopausal women. The plasma NO level was lower in overweight men and postmenopausal women than in normal weight men and postmenopausal women. There were no significant differences in the plasma (b) VEGF or (c) GM-CSF levels among the four groups. There were no significant differences in the plasma (d) TNF-*α* or (e) IL-6 levels among the four groups. Data are given as the mean ± SD. ^∗^*p* < 0.05 versus normal weight.

As shown in Figure 3, the plasma NO level was lower in overweight men and postmenopausal women than in normal weight men and postmenopausal women (*p* < 0.05). There was no significant difference in the plasma NO level between postmenopausal women and age-matched men (normal weight or overweight, *p* > 0.05). There were no significant differences in the plasma GM-CSF, VEGF, IL-6, or TNF-*α* levels among the four groups (*p* > 0.05).

### 3.4. NO, GM-CSF, VEGF, IL-6, and TNF-*α* Secretion by EPCs

**Figure 4:**
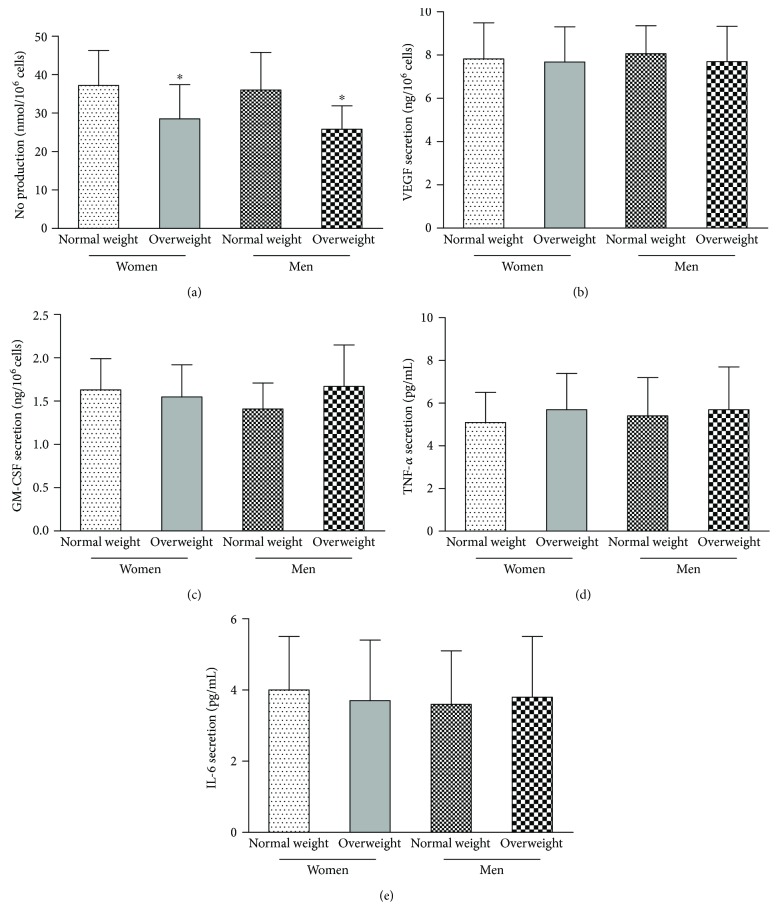
NO, VEGF, GM-CSF, TNF-*α*, and IL-6 secretion by EPCs were evaluated as follows. (a) NO secretion by EPCs in normal weight and overweight men was similar to that in normal weight and overweight postmenopausal women. The plasma NO level was lower in overweight men and postmenopausal women than in normal weight men and postmenopausal women. There was no significant difference in (b) VEGF or (c) GM-CSF secretion by EPCs among the four groups. There was no significant difference in (d) TNF-*α* or (e) IL-6 secretion by EPCs among the four groups. Data are given as the mean ± SD. ^∗^*p* < 0.05 versus normal weight.

As shown in Figure 4, NO secretion by EPCs in postmenopausal women was similar to that in age-matched men (*p* > 0.05). Compared with normal weight men and postmenopausal women, NO secretion by EPCs was lower in overweight men and postmenopausal women (*p* < 0.05). There were no significant differences in GM-CSF, VEGF, IL-6, or TNF-*α* secretion by EPCs among groups (*p* > 0.05).

### 3.5. Correlation between the Migration and Proliferation of Circulating EPCs and Plasma NO Level

**Figure 5:**
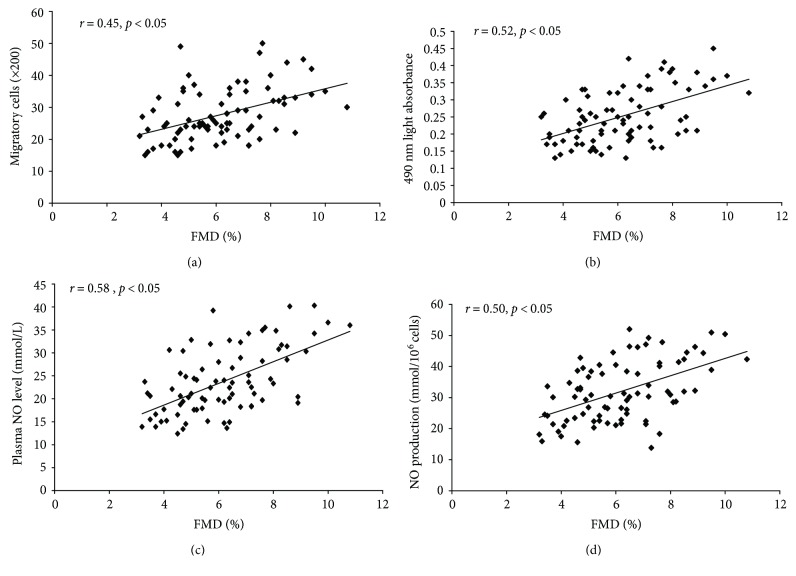
The correlation between the migratory and proliferative capacities of circulating EPCs, and NO level, and FMD was evaluated as follows. There was a correlation between EPC migration (a, b) and FMD. There was also a correlation between the plasma NO level (c) and NO secretion by EPCs (d) and FMD.

As shown in Figure 5, we found a strong unilabiate correlation between the migration and proliferation of circulating EPCs and FMD (*r* = 0.64, *p* < 0.05 and *r* = 0.57, *p* < 0.05, resp.). In addition, there was a significant correlation between the plasma NO level and NO secretion by EPCs and FMD (*r* = 0.68, *p* < 0.05 and *r* = 0.51, *p* < 0.05, resp.).

### 3.6. Expression of Proteins in the CTCPH I/BH4 Signaling Pathway

**Figure 6:**
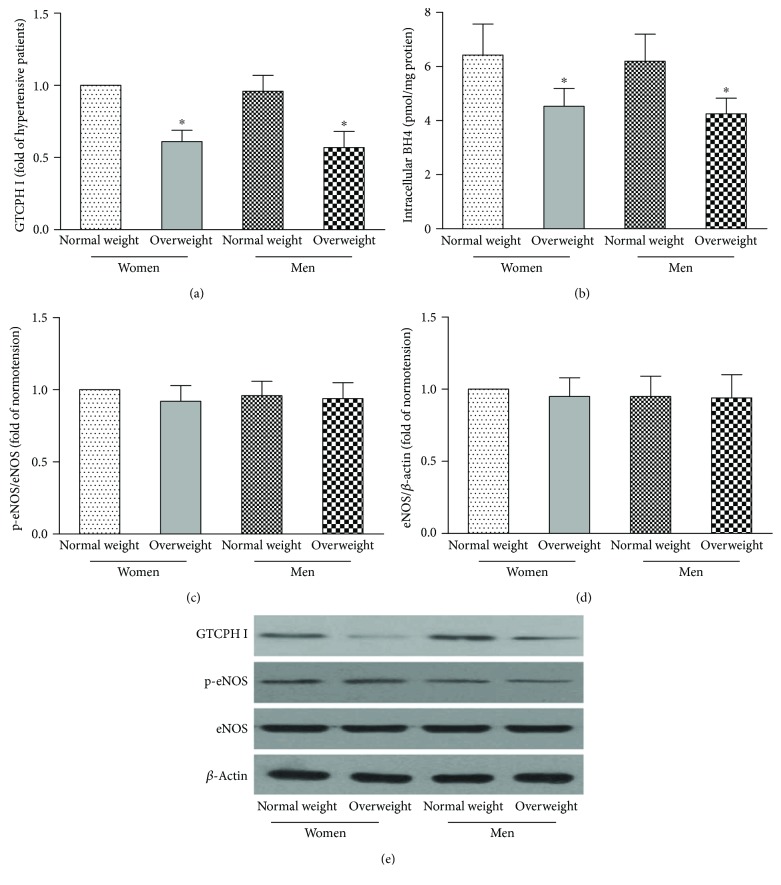
The CTCPH I/BH4 pathway and the phosphorylation of eNOS in circulating EPCs. The levels of GTCPH I (a) and intracellular BH4 (b) in EPCs were lower in overweight men and postmenopausal women than in normal weight men and postmenopausal women. There was no difference in the level of GTCPH I (a) or intracellular BH4 (b) in EPCs between normal weight and overweight postmenopausal women and men. No significant difference was found in either eNOS phosphorylation (c) or eNOS protein expression (d) in EPCs among the four groups. Representative photographs and quantitative analyses of GTCPH I and p-eNOS protein expression in cultured EPCs (e). ^∗^*p* < 0.05 versus normal weight.

To investigate the underlying mechanism of endothelial dysfunction in postmenopausal women, we examined the expression of proteins involved in the CTCPH I/BH4 signaling pathway and the phosphorylation of eNOS in circulating EPCs.  Figure 6 shows that the expression of GTCPH I and intracellular BH4 in EPCs was lower in overweight men and postmenopausal women than in normal weight postmenopausal women and age-matched men (*p* < 0.05). No difference in the expression of GTCPH I or intracellular BH4 in EPCs was observed between normal weight and overweight postmenopausal women and men (*p* > 0.05). In addition, there was no significant difference in either eNOS phosphorylation or eNOS protein expression in EPCs among the four groups (*p* > 0.05).

## 4. Discussion

Obesity-related endothelial injury and dysfunction could significantly enhance the morbidity and mortality of cardiovascular diseases [34]. Estrogen may promote the repair of endothelial injury and deter endothelial dysfunction [35]. The function and number of EPCs in different-aged women are closely correlated with sex hormone levels [36, 37]. Our previous studies suggested that overweight or normal weight premenopausal women had preserved, favorable effects on the function and number of circulating EPCs (date not published). We also observed unfavorable effects among postmenopausal women, regardless of weight, on the function and number of circulating EPCs, suggesting that decreased estrogen may contribute to this phenomenon.

Many studies have shown an increased number of dysfunctional EPCs in postmenopausal women [2, 38–40]. Therefore, we hypothesized that the benefits of circulating EPCs on the endothelium may be abolished in overweight postmenopausal women. In our study, the favorable effects of circulating EPCs were not preserved and the functional activity of EPCs was diminished in overweight postmenopausal women. The functional activity of circulating EPCs in overweight postmenopausal women and age-matched men was lower than that in normal weight age-matched men and normal weight postmenopausal women, indicating that being overweight may lead to endothelial dysfunction and that the function of EPCs is not retained in postmenopausal women.

FMD is considered a reliable and reproducible technique for detecting endothelial function in cardiovascular disease [41]. A previous study showed that endothelial function evaluated by FMD was retained in premenopausal women [10, 15]. However, the phenomenon did not exist in postmenopausal women, indicating that FMD has weaken in postmenopausal women [19]. The results of our study were similar with the previous study. There was a significant correlation between the migration and proliferation of circulating EPCs and FMD, indicating that EPC dysfunction may have contributed to the decreased FMD in the postmenopausal women. In addition, the correlation between the plasma NO level and the capacity of EPCs to secrete NO indicates that decreased EPC function may lead to endothelial dysfunction in overweight postmenopausal women or men. And clinical studies have identified a relationship between increased body weight and cardiovascular disease [7]. This phenomenon suggests that overweight men or postmenopausal women may have a higher risk of cardiovascular events than normal weight men or postmenopausal women.

NO, GM-CSF, and VEGF play crucial roles in regulating the function of circulating EPCs [23, 24]. IL-6 and TNF-*α* induce vascular endothelial damage and play crucial role in regulating function of circulating function [27, 28]. Several studies have demonstrated that overweight or obesity impairs the functional activity of circulating EPCs and diminishes NO bioavailability. The NO level in overweight postmenopausal women was also similar to that in normal weight postmenopausal women, revealing that postmenopause has no beneficial effect on NO production in response to overweight. However, there were no significant differences in GM-CSF, VEGF, IL-6, or TNF-*α* levels among the four groups, suggesting that GM-CSF, VEGF, IL-6, and TNF-*α* may have little relation to overweight-related alterations in circulating EPCs.

Furthermore, the expression of CTCPH I and BH4 in circulating EPCs was reduced in overweight postmenopausal women. Interestingly, the phosphorylation or expression of eNOS in circulating EPCs did not differ in the four groups, indicating that the decreased expression of CTCPH I and BH4 may contribute to the decreased plasma NO level and EPC-secreted NO and that the alteration in eNOS has no effect on the overweight-related decreased activity of circulating EPCs. The loss of circulating EPCs may be attributed to decreased NO production and a downregulated GTCPH I/BH4 signaling pathway. Our data demonstrated that the function and number of circulating EPCs in postmenopausal women were not retained and the mechanism for this phenomenon may reduce NO production by inhibiting the GTCPH I/BH4 signaling pathway. As we know, CXCR4, CXCR7, or PI3K pathway and other related signals play important roles in regulating circulating EPCs and may be potential signaling pathways for improving function of EPCs in overweight postmenopausal women [42, 43]. We will do further study to investigate the underling mechanism in the future.

The present study revealed that the function of circulating EPCs in overweight postmenopausal women was incompetent, indicating that EPCs are crucial biomarkers for evaluating vascular injury in postmenopausal women. Therefore, it is necessary to improve the function of circulating EPCs in postmenopausal women, especially in overweight postmenopausal women, which may be a potential therapeutic target for the improvement of overweight-related endothelial injury. In addition, NO biosynthesis in circulating EPCs in postmenopausal women or age-matched men may be impaired by overweight. NO could encourage the functional activity of circulating EPCs and enhance endogenous endothelial repair capacity.

## 5. Conclusion

To the best of our knowledge, our study is the first to prove that circulating EPCs in postmenopausal women are decreased in number and are dysfunctional, considering the relationship between GTCPH I/BH4 signaling pathway and NO production. Moreover, the GTCPH I/BH4 signaling pathway in circulating EPCs may be a therapeutic target for overweight-related vascular injury in postmenopausal women or age-matched men.

## Figures and Tables

**Table 1:** Clinical and biochemical characteristics.

Characteristics	Normal weight women (*n* = 20)	Overweight women (*n* = 20)	Normal weight men (*n* = 20)	Overweight men (*n* = 20)
Age (years)	56.1 ± 4.7	57.3 ± 4.5	56.7 ± 4.4	55.3 ± 4.1
Height (cm)	163.6 ± 5.6	162.6 ± 6.2	169.3 ± 6.3^#^	167.8 ± 5.0^#^
Weight (kg)	57.7 ± 4.2	70.0 ± 5.6^∗^	63.0 ± 6.5^#^	74.7 ± 4.5^#∗^
BMI (kg/cm^2^)	21.6 ± 1.6	26.5 ± 1.8^∗^	21.9 ± 1.4	26.6 ± 2.3^∗^
Systolic blood pressure (mmHg)	119.4 ± 9.5	120.9 ± 5.9	121.3 ± 6.6	123.3 ± 9.2
Diastolic blood pressure (mmHg)	73.2 ± 6.8	75.0 ± 5.2	74.7 ± 6.3	76.5 ± 6.9
Heart rate (beats/min)	74.8 ± 8.7	75.9 ± 8.5	73.6 ± 8.6	76.6 ± 8.0
AST (mmol/L)	25.0 ± 6.9	26.6 ± 6.3	28.4 ± 4.0	26.3 ± 5.1
ALT (mmol/L)	21.5 ± 5.7	23.5 ± 4.7	24.78 ± 5.6	24.1 ± 6.1
BUN (mmol/L)	5.4 ± 0.7	5.5 ± 0.9	5.1 ± 0.9	5.3 ± 0.7
Cr (mmol/L)	69.1 ± 12.7	71.4 ± 11.6	66.3 ± 14.3	68.1 ± 13.7
LDL (mmol/L)	2.62 ± 0.39	2.76 ± 0.39	2.56 ± 0.46	2.71 ± 0.42
TC (mmol/L)	4.38 ± 0.53	4.54 ± 0.56	4.23 ± 0.63	4.48 ± 0.58
HDL (mmol/L)	1.33 ± 0.20	1.27 ± 0.13	1.29 ± 0.14	1.23 ± 0.12
TG (mmol/L)	1.58 ± 0.19	1.67 ± 0.20	1.63 ± 0.17	1.69 ± 0.16
FPG (mmol/L)	4.34 ± 0.46	4.68 ± 0.56	4.76 ± 0.50	4.54 ± 0.48
Estradiol (pmol/L)	106.0 ± 26.2	116.5 ± 37.0	102.5 ± 20.1	108.1 ± 19.9
FMD (%)	8.72 ± 1.41	7.08 ± 1.26^∗^	8.41 ± 1.24	6.77 ± 1.1^∗^

Note: data are given as mean ± SD. BMI: body mass index; AST: aspartate amino transferase; ALT: alanine transaminase; BUN: blood urea nitrogen; Cr: serum creatinine; LDL: low-density lipoprotein; TC: total cholesterol; HDL: high-density lipoprotein; TG: triglyceride; FPG: fasting plasma glucose; FMD: flow-mediated brachial artery dilatation. ^∗^*p* < 0.05 versus normal weight; ^#^*p* < 0.05 versus premenopausal women.
